# Kidney organoids: steps towards better organization and function

**DOI:** 10.1042/BST20231554

**Published:** 2024-06-27

**Authors:** Jamie A. Davies, Ian Holland, Huseyin Gül

**Affiliations:** Deanery of Biomedical Sciences, University of Edinburgh, Hugh Robson Building, George Square, Edinburgh, U.K.

**Keywords:** disease modeling, metanephros, nephrogenesis, pluripotent, stem cell, tissue engineering

## Abstract

Kidney organoids — 3D representations of kidneys made either from pluripotent or tissue stem cells — have been available for well over a decade. Their application could confer notable benefits over longstanding *in vivo* approaches with the potential for clinically aligned human cells and reduced ethical burdens. They been used, at a proof-of-concept level, in development in disease modeling (including with patient-derived stem cells), and in screening drugs for efficacy/toxicity. They differ from real kidneys: they represent only foetal-stage tissue, in their simplest forms they lack organ-scale anatomical organization, they lack a properly arranged vascular system, and include non-renal cells. Cell specificity may be improved by better techniques for differentiation and/or sorting. Sequential assembly techniques that mimic the sequence of natural development, and localized sources of differentiation-inducing signals, improve organ-scale anatomy. Organotypic vascularization remains a challenge: capillaries are easy, but the large vessels that should serve them are absent from organoids and, even in cultured real kidneys, these large vessels do not survive without blood flow. Transplantation of organoids into hosts results in their being vascularized (though probably not organotypically) and in some renal function. It will be important to transplant more advanced organoids, with a urine exit, in the near future to assess function more stringently. Transplantation of human foetal kidneys, followed by nephrectomy of host kidneys, keeps rats alive for many weeks, raising hope that, if organoids can be produced even to the limited size and complexity of foetal kidneys, they may one day be useful in renal replacement.

## Introduction: what are kidney organoids and why does anyone care?

The term ‘organoid’, used in its modern sense, means a 3-dimensional arrangement of cells that differentiate and self-organize into tissues somewhat representative of the organ that the organoid represents [1]. Organoids are made for three main reasons: (i) to explore tissue and organ development; (ii) to model for disease and to screen possible treatments; and (iii) as technologies for making replacement tissues. Organoids have significant advantages over traditional *in vivo* approaches, because they are accessible and are relatively free of ethical issues; no current organoid would be thought of as being able to experience suffering in a way an organism can (though ethicists are already looking ahead to the possibility that more advanced organoids might [2]). Organoids made from human cells may represent real human organs better than animal models do. On the other hand, organoids show foetal maturity rather than adult, they often include irrelevant cell types [3], and *in vitro* they cannot reflect the full complexity of organs in a body with, for example, an immune system (though, in simple *in vitro* combination experiments, immune cells will interact with them [4]). As with all models, organoids bring both opportunities and limitations; ‘…all models are approximations. Essentially, all models are wrong, but some are useful’ [5].

Kidney organoids, also called metanephric organoids, are constructed for all three reasons outlined above. Mouse kidney rudiments will develop in culture, which has allowed detailed study of renal organogenesis, and organoid technologies allow researchers to follow this development from an anatomical ‘tabula rasa’ with no existing anatomy present [6]. Because kidneys contain transport systems that can concentrate drugs and their metabolites in the cytoplasm, they are a common target for iatrogenic harm; about a quarter of drugs withdrawn in human clinical trials show nephrotoxicity. Having a human organoid-based screen before clinical trials begin would save money and potential suffering, so there is intense interest in this application. Kidneys also suffer from a range of congenital malformations, which might usefully be modeled using organoids. Finally, the kidneys of most mammals are easily damaged by inflammation and regenerate very poorly if at all (the spiny mouse is an intriguing exception [7]). This is fundamentally why so many patients enter renal failure and require dialysis or a transplant. Building new kidneys from stem cells might be one effective treatment if it is ever possible; stem cell-derived organoids are seen by many as a first step [8,9].

## The first kidney organoids

**Figure 1.**
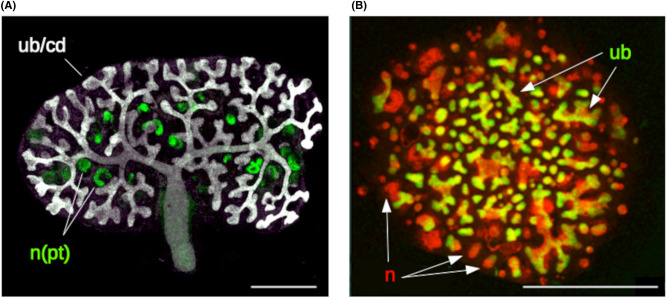
The anatomical/organizational difference between ‘simple’ renal organoids and real foetal kidneys. (**A**) Shows a mouse foetal kidney growing in culture (so that it is as comparable as possible to the organoid). There is a high level of overall organization, with developing nephrons (n) attached to a single ureteric bud/collecting duct system (ub/cd) that exits via a ureter. The green stain, lotus tetragonolobus lectin, shows the proximal tubules (pt) of the nephrons. (**B**) Shows a mouse renal organoid, made from ex-fetu cells: there are developing nephrons (n) and ureteric bud treelets (ub) to which nephrons connect, but no large-scale organization and no way for any urine to exit from the structure. Scale bars are 500 µm. Image sources: (**A**) is original to this review; (**B**) is reproduced with permission from [11], with slight editing to remove part of a text label.

Kidney organoids made entirely by self-organization of suspensions of stem cells, as distinct from recombination of intact separated tissues [10], were first published in 2010 [11]. They used cells isolated directly from mouse embryonic kidneys, dissociated and recombined into random aggregates by centrifugation, cell survival being maintained for the first 24 h by apoptosis-suppressing drugs. The epithelial stem cells from the ureteric bud, the progenitor of the urine collecting duct system, organized themselves into closed epithelia and proliferated to form collecting duct ‘mini-trees’ each with just a few branches. Around these, the mesenchymal cells (nephron progenitor cells and stromal progenitor cells) differentiated into immature nephrons and stroma. When viewed at high magnification, the organoid looked somewhat like a natural developing kidney and had a range of cell types representing most of those in the kidney. When viewed at lower magnification, though, it is obviously not a real kidney (Figure 1A) as it lacks all large-scale organization (Figure 1B).

While this 2010 organoid was a start, there was an obvious need for many improvements, especially to produce human renal organoids, ideally from pluripotent stem cells, and to improve large-scale organization.

## Making renal organoids from pluripotent stem cells

The aim of this section is to illustrate concisely the approaches taken papers that epitomize particular ways of working. Our choice should not be interpreted as a comprehensive review of all work done. The first groups to make renal organoids from pluripotent stem cells all took essentially the same approach, aiming to differentiate the cells first to mesendoderm, then to intermediate mesoderm, then to ureteric bud and/or metanephric mesoderm and on to nephrons and other renal structures. Their efforts were complicated by the fact that the embryonic origin of the ureteric bud is in the nephric duct, which arises in the Pax2+ anterior intermediate mesoderm, whereas the embryonic origin of the metanephric mesenchyme is the Pax2− posterior intermediate mesoderm [12]. Recognition of this caused some groups to try to produce both types of progenitor by a sort of ‘compromise’ protocol that could produce multiple anterior-posterior levels from a still uncommitted stage [13], while others aimed to produce ureteric bud and metanephric mesenchyme progenitor separately, with an intention of recombining them later.

**Figure 2.**
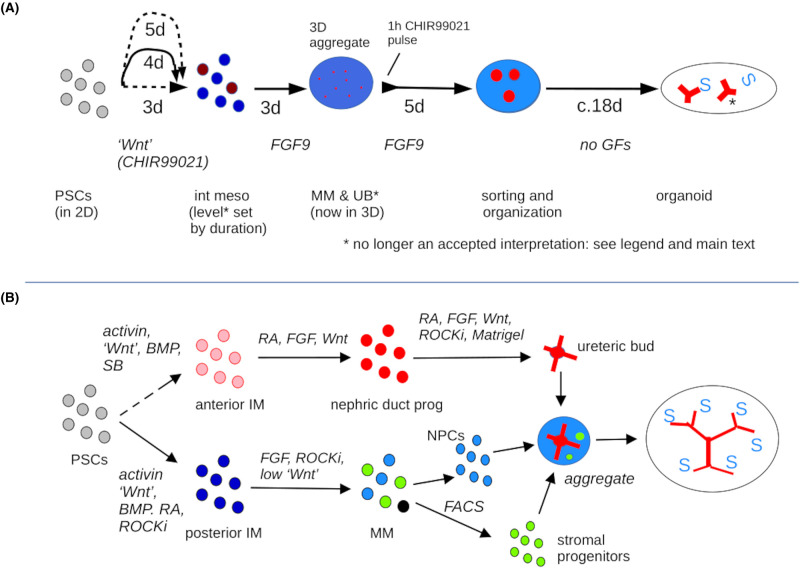
The two approaches to controlled differentiation of pluripotent cells to renal progenitors. (**A**) Depicts the approach of trying to use a single differentiation step to produce all renal progenitor types in a mix, in this case from [15]. At the time of publication, it was believed that different Wnt exposure times near the beginning of the protocol could set the ratio of GATA3+ ureteric bud cells and nephron progenitor cells. It has since become clear that the GATA3+ cells were probably distal nephron, and that the Wnt exposure is setting degree of distalization [16,17]. (**B**) Depicts the approach of making the ureteric, nephron and stroma progenitor cells separately and then recombining a single ‘ureteric bud’ with the other progenitor types. The method shown is an amalgam of [18] and [19], and some arrows represent more than one step in terms of medium changes. This figure illustrates only the cell types wanted: in reality, other cell types also form.

The first approach, attempting to produce all progenitors from the same differentiation protocol, is exemplified by the work of Melissa Little's lab. They first developed a protocol for differentiating human embryonic stem cells in a series of steps, to primitive streak (using the wnt agonist CHIR99021) to intermediate mesoderm (using FGF9) to metanephric mesenchyme and ureteric bud (using BMP4 and activin A, followed by FGF9, BMP7 and retinoic acid). These cells interacted to form very immature nephrons (renal vesicles) [14]. Two years later, the same group published a refined protocol that used human induced pluripotent cells and produced organoids with more mature nephrons and apparently some ureteric bud (GATA3+) cells. The ratio between GATA3+ and other elements could be chosen by varying the length of exposure to CHIR99021 [15] (Figure 2A). Later single cell sequencing analysis suggested, however, that many of the GATA3+ cells might in fact be distal tubule [16,17], though surprisingly these cells can transdifferentiate into ureteric epithelium if cultured with WNT, FGF and GDNF *in vitro* [20], something not expected from normal development.

The second approach, of making the progenitor types separately (Figure 2B), saw its first prominent success in work by Joseph Bonventre's group who published a differentiation protocol to make human nephron progenitor cells [18]. The project of producing different types of stem cell separately and then combining them to make complete kidney organoids is exemplified by the work of Ryuichi Nishinakamura's lab. They established the posterior origin of the metanephric mesenchyme, and designed a stepwise differentiation protocol using BMP and Wnt signalling to lead both mouse and human pluripotent cells to become posterior mesenchyme (T+, Hox11+), reduced Wnt and added activin and retinoic acid to differentiate the cells to posterior intermediate mesoderm (Osr1+, Hox11+), then used FGF9 to differentiate these cells to nephron progenitors (Six2+, Hox11+) and Wnt4 to differentiate them into nephrons [12]. They then developed a separate protocol to differentiate pluripotent cells into ureteric bud progenitors. This involved a shorter incubation time in Wnt, with no activin and with drug-mediated Smad inhibition, to differentiate mesendoderm into anterior intermediate mesoderm. Wnt, retinoic acid, and FGF9 then promoted differentiation into nephric duct progenitors [18]. Retinoic acid, Wnt and FGF9 and GDNF differentiated these into ureteric bud (again, this works for mouse and human), and these buds would produce organotypic branched trees when combined with natural metanephric mesenchyme, or with a mixture of pluripotent cell-derived nephron progenitors and ex-fetu stromal progenitor cells. Our development of a method to make the stromal cell progenitors too, from pluripotent cells, and combination of these with nephron progenitors and ureteric bud progenitors, resulted in an all-mouse-pluripotent-cell-derived kidney organoid arranged around a branched ureteric bud system [19].

Opinions about the relative merits of these two approaches can be strongly polarized. Careful single-cell RNAseq analysis has shown that different approaches can produce a very similar set of cells (including cells that should not be present), and similar dendrograms of cell types [16]. The main difference is how well the ureteric bud lineage is represented, and the balance of different nephron cell types. Which is ‘best’ depends on the application, and we make no recommendation beyond potential users studying the merits of each, and making their own decisions. The authors’ lab uses both types of protocol, for different purposes [19,21].

The problems of extraneous cell types, unresolved after a decade's work, may be warning us that the whole approach of trying to make a single organ directly from pluripotent cells is an over-ambitious aim. Very advanced models of early human embryos can be made from pluripotent cells, including axioloids that show extended trunks, somitogenesis etc. [22]. It may be that advancing these embryo models to the stage from which kidney rudiments can be taken, as they have been taken from mouse embryos for decades, will be a route to much more pure and realistic *in vitro* models for human kidney.

## Developing organoids for specific pathological and pharmacological applications

Both of the 2015 papers describing renal organoids made from pluripotent cells [15,16] demonstrated the potential of organoids to screen drugs, showing that cisplatin and gentamicin, known proximal nephrotoxicants, were toxic to proximal tubules in the organoids. The same year, organoids were being generated from genetically modified human pluripotent cells. CRISPR gene editing was used to introduce truncating mutations in the genes PKD1 or PKD2, mutation of which is associated with autosomal polycystic kidney disease. The result was the formation of cysts in the organoids, but not in wild-type controls [23]. Gene editing and other genetic engineering techniques have been applied to pluripotent cells before making them into organoids to model a variety of genetic diseases including karyomegalic interstitial nephritis [24], apolipoprotein risk variants [25] and nephronophthisis-related ciliopathies [26]. Use of patient-derived pluripotent cells is an alternative to gene editing. Examples include study of IFT140-associated ciliopathy in the etiology of nephronophthisis [27], Finnish-type congenital nephrotic syndrome [28], Fabry disease [29], polycystic kidney disease [30], Alport syndrome [31], and karyomegalic interstitial nephritis [24]. Correction of the relevant mutation in patient-derived cells is important to show the pathology really is caused by that mutation and not something else.

The use of organoids for toxicity screening requires a method for assessing damage, and the faster and more convenient this is, the higher throughput the screen can be. To this end, human induced pluripotent cells have been engineered to report oxidative stress fluorescently. In a challenge with a panel of blind-coded compounds supplied by an industrial collaborator, renal organoids made from these cells were able to identify those known to be toxic to kidneys [21].

The use of organoids in modeling and screening applications is, however, still limited by their shortcomings. One is maturity: the epithelia of renal organoids mature to mid-late foetal stages at best, and this affects aspects of their physiology, potentially including transport [15]. Significant numbers of transporters (e.g. BK_Ca_, CFTR, Cubulin, ENaC, Megalin, MRP OAT1,, OCT1, OCT2, Megalin, MRP, ROM-K, V-ATPase) are present in mouse ex-fetu organoids and in those derived from mouse and from human pluripotent cells, and they show drug-inhibitable transport activity [32–37]. More work needs to be done to compare transport and substrate concentration to *in vivo* kidneys before it is clear how well they will predict pharmacokinetics and pharmacotoxicity seen in real kidneys. A second limitation is lack of glomerular filtration and consequent lack of filtrate/urine flow. Podocytes, the cells that make the filter, do form in organoids and so do small blood vessels, but they are not connected to large blood vessels that could be connected to a pump to simulate blood circulation. A third is lack of a full immune/inflammatory system, which is important to some types of renal damage. These issues overlap strongly with those that prevent current organoids being a practical basis for regenerative medicine. One possible way to circumvent some of these problems is to engraft human organoids representing a renal disease into a wild-type host animal; this approach has been used successfully to explore the significance of deficiencies in the renin-angiotensin system [38].

## Improving the anatomical realism of organoids

The most obvious difference between the organoids described above and foetal kidneys is that large-scale organization is essentially absent from the organoids. Real kidneys are organized, in terms of both their development and their final anatomy, around the collecting duct tree. This radiates from the ureter ‘trunk’ and the renal pelvis, through the medulla, to the finest branches out in the cortex. The nephrons that survive to birth form near, and attach to, these outer branches, and send loops of Henle down into the medulla. The blood vessel system enters the kidney beside the ureter and radiates through the kidney following its collecting duct tree (both developmentally and anatomically), to reach the proximal poles of the nephrons. Having made a glomerulus there, blood vessels leave to follow the loop of Henle as it dives down to the medulla and back to make a second capillary system (the vasa recta), before exiting via the venous system.

**Figure 3.**
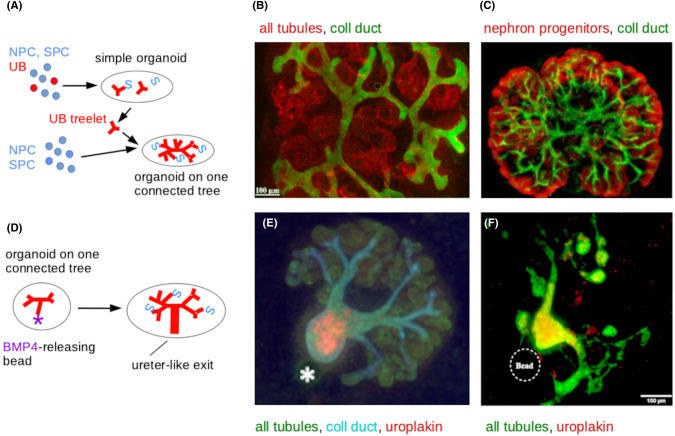
Methods to make better organize organoids. (**A**) Outline of sequential organoid method to arrange the organoid around a single collecting duct tree; NPC and SPC are nephron and stromal progenitor cells respectively, and UB ureteric bud cells. (**B**) An organoid made this way from ex-fetu mouse renogenic stem cells [39]; (**C**) an organoid made this way from mouse pluripotent cells [40]. (**D**) Outline of a method for inducing a ureter-type exit in organoids made as in (**A–C**); (**E**) an organoid made this way from ex-fetu renogenic stem cells; (**F**) an organoid made this way from mouse pluripotent cells. Image sources: (**B**) is by Veronica Ganeva, JAD's lab, (**C**) is from [40], (**E**) from [41], (**F**) from [19], all reproduced under their CC BY 4.0 licences (https://creativecommons.org/licenses/by/4.0/).

The primacy of the collecting duct system in organizing the kidney made this a natural focus for improving organoids. The first step was replacing multiple collecting duct treelets with one single tree. This was done by making an organoid, isolating one collecting duct treelet from it, then combining this with nephron progenitor and stromal progenitor cells only. This was achieved in 2011 for organoids made from ex-fetu murine cells [39] (Figure 3A,B), and in 2017 for organoids made from pluripotent human cells [18]. The quality of organoids derived from mouse pluripotent stem cells was improved further by differentiating nephron progenitor cells and stroma progenitor cells separately, and then combining them with one another and with a collecting duct progenitor isolated from a previous organoid [19,40] (Figure 3C). Presumably this will be tried with human pluripotent cells soon.

Organoids produced around one collecting duct tree are more realistic than basic organoids but the tree has no exit. This problem was again first addressed using ex-fetu mouse renal progenitor cells. Slow-release beads were used to make a locally high concentration of BMP4 near one branch of a young collecting duct system in an organoid, to mimic signalling conditions around a natural developing ureter [41] (Figure 3D). This one branch branched no more but thickened and expressed proteins characteristic of ureter, effectively becoming the trunk of the tree. The resultant organoid (Figure 3E) was now very similar to an early foetal kidney. The idea of using local BMP4 to induce the ureter was later combined with that of making organoids from one pluripotent cell-derived collecting duct and separate pluripotent cell-derived nephron and stroma progenitor cells. This produced an all-pluripotent cell-derived organoid with one collecting duct tree and ureter-like exit [19] (Figure 3F), but it was not as realistic looking as the one from ex-fetu mouse cells (Figure 3E). This is been a continuing theme of the field, and suggests that there is still room for improvement of differentiation protocols from pluripotent cells. It is one reason that we tend to pioneer techniques with organoids made from ex-fetu renal stem cells, and only then try to achieve the same thing from pluripotent stem cells.

One possible approach to improving organoids is to add further layers of specific, external control, beyond slow-release beads, to the basic system of self-organization. A potentially far more accurate control method is optogenetics. Here targeted light stimulation, down to a single cell resolution, of wavelength-responsive proteins can regulate gene expression [42] and therefore control the local regulation of morphogens or differentiation processes. Applying such methods to kidney organoids may ultimately allow the spatiotemporal control of key developmental events and potentially lead to more mature tissue. However, the current use of this technology in any organoid type is limited and will require extensive future optimization to apply it towards kidney tissue.

Further development of all organoids described above, in terms of maturation and also growth, is limited by diffusion of oxygen, nutrients and metabolites in 3D culture. In natural kidneys, the diffusion problem is solved by blood circulating in vessels that permeate the tissue. This makes adding vasculature to organoids a critical next step in producing something more realistic and functional. It is a problem for many organoids, not just kidneys.

## The problem of the vasculature

Kidneys’ main task is excretion, which depends on circulating blood reaching glomeruli; knots of leaky capillaries surrounded by the podocytes at the proximal end of the nephron. What is more, recovery of water depends on a precise relationship between blood vessels and nephrons, so that their loops of Henle maintain a hypertonic medulla. Moving from organoids to a properly functioning small kidney therefore demands not just ‘vasculature’, but the development of a vascular system with a specific anatomy.

There was initial excitement that capillary endothelia appeared in organoids made from pluripotent cells formed extensive networks [19]. These had little relationship to the collecting duct tree, even when there was such a tree, and vessels above the scale of capillaries were absent. This was not surprising; the natural vasculature of the kidney invades from the outside and follows the collecting duct tree from ‘trunk’ outward along its branches [43], branching as it does to transition from renal artery to segmental arteries to arterioles and capillaries. Simply adding endothelia, such as HUVEC cells, to the ureter pole of a kidney in culture (even an ex-fetu one) does not result in recapitulation of that natural tree. If a more mature kidney, already having these vessels, is removed from a mouse foetus and cultured, the large vessels degenerate though the capillaries prosper [44,45]. Treatment with pro-angiogenic factors does not ameliorate this. The most likely reason is the well-known requirement of larger blood vessels for blood flow to maintain their survival [46], a feature that allows vascular systems to remodel in response to blood flow itself. While some organoid culture systems have been developed that use ‘flow’ and claim better maturation [47] this is primarily flow of medium *around* the organoids, not flow through vessels: tracer beads from the flowing medium can enter some of these vessels and move within them. In a broadly similar system [48], tracer lectins in the bulk flow took around 11 min to reach the first glomeruli. It is arguably impressive that lumenal flow occurs at all under these conditions, but the flow and architecture clearly remain a long way from the blood flow and vasculature architecture of an *in vivo* kidney. Again, capillary-scale vessels thrive but large vessels do not form.

The most obvious and life-like solution to the problem of vascularization with flowing blood is transplantation. The first transplanted renal organoids were published in 2012 [49]. These were basic organoids made from ex-fetu mouse renal stem cells, transplanted under the kidney capsule of an adult rat. They survived and were vascularized by host vessels carrying flowing blood, that branched within the graft. Organoids of this type have multiple collecting duct treelets, not a single tree, so alignment of the vessels with the collecting duct tree was not an accessible question. At least some glomeruli formed and were served with flowing blood, and tracer passed from the blood into the urinary space. Broadly similar transplantation experiments, to a variety of hosts and sites, have since been done with organoids made from pluripotent cells [50–55]. These have generally been implanted intact but have sometimes been implanted as dissociated cells to form organoids *in situ* [56]. In all cases, host-derived vascularization was observed, sometimes connecting to graft-derived capillaries, including formation of glomeruli, and nephron maturation was enhanced though no system reached full maturity. In all cases, the organoids used were simple (not based on a single collecting duct tree with an exit), so there was no realistic reference renal anatomy against which realism of vascular anatomy could be judged. Transplanted organoids made from pluripotent cells also showed problematic features, such as inappropriate stromal expansion [55]. This again suggests that more work needs to be done in differentiating pluripotent cells to precisely the correct cell type/state to represent natural renal progenitors (the problem is not seen when ex-fetu material is used). The realism of host-derived vasculature could perhaps be assessed if the more advanced organoids described in the previous section, with a single collecting duct tree and ureter-like exit, were used.

Vascularization of *in vitro* organoids might be done several ways. If anatomically realistic organoids contain guidance cues for vessels, then an *in vitro* system of parallel arterioles and venules, with pumped blood, and the organoid cultured between them, may be sufficient to induce ‘natural’ invasion of vessels. We made some attempts to do this, but the system was difficult to set up and, while vessels entered the kidney, the resulting vasculature was far from normal [57]. Alternatively, it might be possible to guide growing vessels from external endothelia ‘manually’ using, for example, optogenetics to steer their leading edges. The need for flow will still be a problem for large vessels.

## Models for transplanting organoids for functional replacement of kidney

If their problems can be solved, organoids will effectively be identical with foetal kidneys. A simple test of whether they will be useful for renal replacement is to ask whether natural foetal kidneys can be transplanted into adult hosts and, after growth, substitute for the host kidneys. Foetal kidney transplantation has a long history, outside the scope of this review. The most successful attempt transplanted 17–18 week human foetal kidneys into rat hosts and used a device to reduce the blood pressure experienced by the graft [58]. The graft grew and acquired new nephrons. After 30 days, the host kidneys were removed. Control nephrectomized rats survived 3 days, those with the human graft survived a mean of 122 days with a maximum of 304 days. This was not a perfect outcome (normal rats live longer) but, given the complications of interspecies transplantation, it is a promising indication that having organoids accurately representing even the foetal stage of development might be enough to replace renal function. This may be especially useful for congenital diseases in which the later need for renal replacement can be predicted before a patient becomes really ill.

## Prospects for partial replacement

One alternative to replacing complete kidneys with organoid-derived replacements would be replacement of areas of damaged renal tissue. At first sight, this approach seems unlikely because of the multiple ‘plumbing problems’ involved for blood and urine. Grafting a simple organoid into a host kidney does not result in this connection, possibly because of intervening fibrous tissue [59]. Nevertheless, recent publications suggest that appropriate junctions might self-organize. Collecting ducts and ureter-like tubes made from mouse pluripotent stem cells will connect to collecting ducts or ureter respectively, of host mouse foetal kidneys in culture [60]. It may be possible to trigger this healing response using soluble factors, and at least one group has set out to do this, using an elegant species-sensitive assay to detect the presence of products of human nephrons in mouse (host) urine [59]. There is also intense research into treating damaged kidneys with stem cells (as a suspension of stem cells, rather than organoids made from them): that is beyond the scope of this article but is the topic of recent reviews [61–63].

## Conclusions

Basic kidney organoids containing a full complement of renal epithelia are relatively easy to make, and have shown promise as models of disease or drug/toxin response. More complex techniques make overall organoid anatomy more realistic, though organoids made from pluripotent cells still show aberrant features (non-renal cell types, overgrowth of stroma). Normal vascular systems are missing from organoids *in vitro*; capillaries form but larger vessels do not. Transplanted into hosts, organoids gain host-derived vasculature that connects to glomeruli and gives some function: the organoids so far transplanted have not been anatomically realistic enough for tests of realism of vasculature or of full function. Foetal kidneys transplanted into adult hosts show promising function, providing a model for transplantation of better organoids. The most pressing need now is transplantation of more realistic organoids to test whether host vasculature enters them and makes appropriate vascular anatomy, and to assess function. Evidence is also growing that partial replacement of damaged renal tissue with organoid-derived material might be an alternative to replacing whole kidneys.

## Statement about AI/LLM use and image alteration

Neither the research for nor the writing of this article made any use of large language models or other AI assistance. The edges of the images in Figure 1 were slightly altered to black out scale bars and a badly placed label, so that the style and placement of new scale-bars and labels was uniform. These manipulations took place only on the area of the black background and did not impinge on the area occupied by any biological sample.

## Perspectives

1. Kidney organoids are important for developmental research, disease modeling, drug screening and, potentially, as a route to renal regeneration and replacement.
2. Current organoids represent urine-carrying tubules and stroma of small foetal kidneys, with some function and some vasculature, but realistic vasculature is missing and organoids made from pluripotent cells usually contain some aberrant cell types.
3. Work is needed to differentiate pluripotent cells to only the correct types, and to assemble a properly organized vascular system, which may allow much better *in vitro* growth and better *in vivo* function.

## Open Access

Open access for this article was enabled by the participation of University of Edinburgh in an all-inclusive *Read & Publish* agreement with Portland Press and the Biochemical Society under a transformative agreement with JISC.
